# Women Workers

**Published:** 1900-11-03

**Authors:** 


### The Hospital

A Journal of

The Medical Sciences and Hospital Administration.

Vol. XXIX.—No. 736.] Edited by Sir Henry Burdett, K.C.B., and Solomon C. Smith, M.D., M.R.C.P. [November 3, 1900.

### Women Workers.

A body which describes itself as the "National Union of Women Workers" has devoted no less than three entire days of last week to a "Conference" at Brighton, and debated there on a great variety of questions, most of which were treated in a somewhat discursive manner. The Conference seems to have been largely attended, for the space beneath the dome of the pavilion is said on more than one occasion to have been densely crowded, most notably, perhaps, when Mrs. Humphry Ward read what was manifestly a highly interesting paper on the educational treatment and capabilities of crippled children. Neither this subject, however, nor the majority of those which preceded or followed it appears to us to appertain in any exclusive way to women; and, in so far as we have been able to judge from the reports in the *Times* paper, the discussions themselves were of a kind more likely to proceed from amateurs than from workers. Possibly the magnitude of the audiences may in some measure be explained by the ordinary abundance, in Brighton, of women who, like frozen-out gardeners, "have no work to do," and for whom an opportunity of listening to speeches would be a temptation too great to be resisted. We are not of the number of those who regard feminity as synonymous with incapacity, but we are equally far from regarding it as synonymous with capacity, and we think it quite as necessary for a woman as for a man that a business should be learnt before endeavours are made to teach it. The business of diminishing the evils wrought by intemperance, for example, is one almost equally calculated to tax to the uttermost the best faculties of a statesman, and to display to the uttermost the imbecility of a platform orator, whether male or female. On this subject, of course, the ladies were eloquent in their exhibition of ignorance of the difficulties of the subject, and in their headlong advocacy of remedies which would be worse than the disease. Fortunately for the kingdom, as we have lately had occasion to observe, the sceptre has fallen from the hands of faddism, if not in perpetuity, at least for a considerable season; and there will be ample opportunity for reasonable and responsible people to initiate social legislation founded upon wisdom and experience.

We need hardly say that no one can esteem the real work of women more highly than we do; and, for that very reason, we have but slender patience with the irresponsible chatter of some who profess to be their advocates or their representatives. It would be difficult to compress into a few lines a greater amount of folly than was uttered in the course of a so-called debate on the "work of women on hospital and other boards." A lady from Southwark declared that in hospitals there was a great tendency to look on patients as "mere cases;" and a lady from Leicester said that women on hospital boards would refuse to consider hospitals as schools for students. They would consider them primarily as places where sick people were to be cured. Now, in the first place, it is obvious that, as far as physicians and surgeons are concerned, nothing could be less desirable than that they should take an emotional view of the already complicated problems which they are called upon to solve. To do so would necessarily impair the soundness of their judgments and injuriously affect the steadiness of their nerves. It is perhaps equally obvious that the instruction of students is a far more important part of hospital work, measured by its value to the public, than the cure of the actual inmates of the wards. A patient has, let us say, typhoid fever. His life is of high importance to his family; and it is admitted on all hands that nothing should be done by which his prospect of recovery can be jeopardised or diminished. The benefit, nevertheless, is limited to a narrow circle. But if his illness is made a means by which fifty students are taught how to conduct a case of typhoid fever to a successful issue the consequent benefit is far more widely diffused, and may extend to countless families and to all parts of the globe. Moreover, hospitals could not be efficiently conducted without the aid of students, who perform gratuitously, for the sake of instruction, and often with the greatest possible zeal and enthusiasm, an immense amount of necessary work which would otherwise require the employment of paid officials. Not only do they do this, but they also supply, to a very great extent, that element of personal sympathy the absence of which the Southwark lady probably intended to assert and to deplore. Everyone who is conversant with the inner life and working of a hospital knows how much patients will impart to students—who are on duty in relation to them as clinical clerks or dressers—of their anxieties and their affairs, and how often a surgeon will depute his dresser to explain to a patient the character of an operation, the necessity which exists for its performance, and the benefits which it may be expected to secure. The ladies from Southwark and from Leicester succeeded in betraying the most absolute want of knowledge of the matter on which they talked, and they succeeded in nothing else.

